# Health-Promoting Properties of Pectin–Polyphenol Complex Extracted from Olive Oil By-Product Alperujo: Antioxidant, Antiproliferative, and Anti-Inflammatory Activities

**DOI:** 10.3390/antiox13091066

**Published:** 2024-08-30

**Authors:** Alejandra Bermúdez-Oria, María Luisa Castejón, Fátima Rubio-Senent, Guillermo Rodríguez-Gutiérrez, Juan Fernández-Bolaños

**Affiliations:** Department of Food Phytochemistry, Instituto de la Grasa (Spanish National Research Council, CSIC), Pablo de Olavide University Campus, Building 46, Ctra. de Utrera km. 1, 41013 Seville, Spain; mcastejon1@us.es (M.L.C.); f.r.senent@csic.es (F.R.-S.); guirogu@ig.csic.es (G.R.-G.)

**Keywords:** olive oil by-products, alperujo, pectin–polyphenol complex, antioxidant activity, antiproliferative effects, inflammation, cytokines, macrophages

## Abstract

This research explores the health-promoting properties of the pectin–polyphenol complex extracted from alperujo, a by-product of olive oil production. This study investigates the chemical composition and antioxidant activity of the extracts, revealing their high antioxidant activity in vitro. Cell viability assays conducted on colon carcinoma cells (Caco-2) demonstrate the inhibitory effect of the extracts on cell proliferation. However, the extracts do not affect the viability of differentiated Caco-2 cells, suggesting a selective antiproliferative action. Additionally, the extracts reduce intracellular reactive oxygen species (ROS) and nitrite (NO) production in LPS-stimulated murine peritoneal macrophages. Furthermore, the extracts exhibit anti-inflammatory effects by downregulating the secretion of pro-inflammatory cytokines TNF-α, IL-1β, and IL-6 in these macrophages. These findings highlight the potential of pectin–polyphenol complexes as functional ingredients with significant health benefits, demonstrating antioxidant, antiproliferative, and anti-inflammatory properties.

## 1. Introduction

Pectins are complex anionic polysaccharides found in the cell walls of higher plants and are a component of soluble dietary fiber with an average molecular weight between 50 and 150 kDa [1]. Although human digestive enzymes cannot fully process pectins, they are fermented by colon bacteria [2]. Pectins offer numerous health benefits, including reducing the risk of type 2 diabetes [3], cancer [4,5], obesity [6], and inflammatory bowel diseases [7,8]. Traditionally, these benefits have been linked to their prebiotic effects and the stimulation of short-chain fatty acid production [9]. However, there is evidence that soluble fiber directly interacts with the immune system [10]. Pectins are composed of galacturonic acid with various neutral sugar side chains. Their physicochemical properties and physiological responses, such as anti-inflammatory abilities, depend on factors like chain length, degree of methylation, branching pattern, and molecular weight [7,10]. Pectins are commonly sourced from citrus and apple wastes, but our research has identified alperujo, an olive oil by-product, as a promising alternative. Olive waste, known as olive pomace or alperujo, is a by-product generated in large quantities during the production of olive oil using a continuous two-phase extraction system. This process results in both oil and a mixture of solid residue and vegetation water, forming a semifluid paste. Alperujo consists of olive vegetative water and solids, including skin, seeds, pulp, and fragments of olive stones. Rich in beneficial components found in olive fruit, alperujo is a valuable source of compounds such as phenols, sugars, cell wall polysaccharides (including pectin, cellulose, and hemicelluloses), proteins, tocopherols, carotenoids, and chlorophylls [11]. Despite its richness in bioactive compounds, the primary uses of alperujo are currently limited to animal feed, composting, and bioenergy production [12]. However, steam treatment of alperujo provides an alternative method for extracting these bioactive compounds, as in this case, for the production of pectic extracts [13].

The positive health effects of polyphenolic-rich plant foods are well known, such as antioxidant and anti-inflammatory activities, as well as other biological functions of polyphenols [14]. Recently, we have shown that the formation of these pectin–antioxidant complexes occurs during the milling of the olives and the slow beating of the resulting paste (malaxation) that is carried out for olive oil extraction. These crushing and beating procedures break the cells, releasing phenols from vacuoles and also facilitating the interaction between polysaccharides from the cellular wall and hydrophilic compounds, including phenols and proteins [15]. The presence of significant amounts of phenolic compounds bound to pectin is responsible for a lot of important activities, such as significant antioxidant and antiproliferative properties as determined using different human cancer cells (colon carcinoma Caco-2; monocytic leukemia THP-1; and bladder cancer RT112, T24, J82; SCaBER cells), without affecting the growth of confluent Caco-2 that resemble healthy cells [12,16,17]. However, research on extracts obtained from olive waste, composed of polyphenols associated with pectic polysaccharides, has shown great variability in terms of antiproliferative and antioxidant activity [18]. It should be emphasized that the polyphenol content of each material, as well as its interaction with pectin, varies depending on several factors, such as olive variety, climatic conditions, agricultural practices, ripening stage, olive storage time, and processing techniques employed [12].

The objective of this study is to investigate and characterize the health-promoting properties of the pectin–polyphenol complex extracted from olive oil by-product alperujo. Antiproliferative activities were carried out in the Caco-2 cell line. We also aim to investigate possible anti-inflammatory effects using an ex vivo inflammation model of mouse peritoneal macrophages stimulated with bacterial lipopolysaccharide (LPS). Macrophages represent one of the most important cell populations of the immune system, acting in both innate and adaptive immunity and playing a fundamental role in the inflammatory process [19]. This anti-inflammatory activity of the pectin–polyphenol complex will be evaluated by quantifying the levels of inflammatory mediators such as intracellular nitric oxide (NO) and reactive oxygen species (ROS) and the modulation of pro-inflammatory cytokines TNF-α, IL-1β, and IL-6 in LPS-induced inflammatory responses in murine peritoneal macrophages.

## 2. Materials and Methods

### 2.1. Material

Galacturonic acid (≥97%), m-hydroxydiphenyl (90%), Folin–Ciocalteu reagent, azobis (2-amidino-propane) dihydrochloride (AAPH) (97%), fluorescein, L-rhamnose, L-arabinose, D-galactose, D-glucose, D-mannose, D-xylose, and myo-inositol (≥99% for all sugars) were obtained from Sigma-Aldrich (St. Louis, MO, USA). Caco-2 cells were provided by the European Collection of Authenticated Cell Cultures, Public Health England. Cell culture media and serum were from Gibco, Thermo Fisher Scientific (Waltham, MA, USA). Thioglycollate solution was from (BD Difco™ Dehydrated Culture Media: Fluid Thioglycollate Medium, Le Pont de Claix, France).

### 2.2. Raw Material

The olive waste (alperujo) (a semi-solid residue composed of olive peel, pulp, seeds, and ground stones) was collected at the beginning of the harvest season and supplied by a pomace oil extraction factory (Marchena, Seville, Spain) after a certain period of storage of the paste.

### 2.3. Pectin–Polyphenol Extracts

Alperujo (20 kg), the primary by-product of olive oil production, was subjected to hydrothermal treatment at 80 °C for 60 min with 3% citric acid. The treated material was then centrifuged at 4700× *g* (Comteifa, S.L., Barcelona, Spain) to separate the solid and liquid phases. The liquid phase was ultra-filtered using a 3 kDa membrane. The fraction larger than 3 kDa was precipitated in 80% ethanol and allowed to dry.

### 2.4. Characterization of Pectin–Polyphenol Extract

Galacturonan (anhydrogalacturonic acid) content was measured using the m-hydroxydiphenyl method for uronic acids as described by Blumenkrantz and Asboe-Hansen (1973) [20]. Neutral sugars were analyzed following hydrolysis with 2N trifluoroacetic acid (TFA) at 121 °C for 1 h. Hemicellulosic sugar composition was determined by reducing and acetylating solubilized sugars, followed by gas chromatography (GC) (Hewlett-Packard (HP) 6890 Series GC System, Palo Alto, CA, USA) analysis of the resulting alditol acetates, using inositol as an internal standard, according to the method by Englyst and Cummings (1984) [21]. Calibration was performed with standard solutions of L-rhamnose, L-arabinose, D-galactose, D-glucose, D-mannose, and D-xylose. The chromatographic conditions were described by Lama-Muñoz, Rodríguez-Gutiérrez, Rubio-Senent, and Fernández-Bolaños (2012) [22]. Total phenolic content was determined using the Folin–Ciocalteu spectrophotometric method and expressed as grams of gallic acid equivalents, as described by Singleton and Rossi (1965) [23].

### 2.5. Antioxidant Activity

#### 2.5.1. Oxygen Radical Absorbance Capacity (ORAC) Assay

The ORAC assay measures antioxidant capacity by assessing the inhibition of oxidation triggered by peroxyl radicals, which are formed through the thermal breakdown of 2,2′-azobis (2-amidino-propane) dihydrochloride (AAPH). The reactive oxygen species (ROS) generated during this process cause a reduction in the fluorescence signal of the probe fluorescein. The antioxidant capacity of the samples was evaluated using the method outlined by Ou, Hampsch-Woodill, and Prior (2001) [24], with minor modifications. The sample was diluted with sodium phosphate buffer (10 mM, pH 7.4), and 25 μL of the sample was transferred to a microplate. Blank wells received 25 μL of phosphate buffer, while the standards received 25 μL of Trolox solutions (10–140 μM). Then, 150 μL of 1 μM fluorescein was added to all wells. After a 15-min incubation at 37 °C, 25 μL of 250 mM AAPH was added to each well to initiate the reaction. Readings were taken every 5 min for 90 min using a microplate reader (Fluoroskan Ascent™, Thermo Scientific™, Waltham, MA, USA) at excitation and emission wavelengths of 485 nm and 538 nm, respectively. The results were calculated based on the difference in the areas under the fluorescein decay curve between the blank and the sample and expressed as μmol Trolox equivalents/g of the sample.

#### 2.5.2. Antiradical Activity: 2,2-Diphenyl-1-picrylhydrazyl (DPPH)

The free radical-scavenging activity was assessed using the DPPH method, as detailed in a previous study [25]. This method evaluates the ability of antioxidants to neutralize the stable radical DPPH•. Absorbance measurements were taken using an iMark microplate reader model 550 (BioRad^®^, Hercules, CA, USA). DPPH• exhibits an absorption peak at 515 nm, which diminishes when reduced by an antiradical compound. The assay results were calibrated with Trolox, and a calibration curve was generated by plotting absorbance at 515 nm against known concentrations of Trolox, which was expressed as μmol Trolox equivalents/g of the sample.

### 2.6. Caco-2 Cell Culture

Colon carcinoma cell lines (Caco-2) were maintained in 10 cm dishes with a 5% CO_2_ atmosphere using Dulbecco’s Modified Eagle Medium (DMEM) containing 1000 mg/mL glucose, 110 mg/mL pyruvate, and 580 mg/mL glutamine. The medium was supplemented with 10% fetal bovine serum, 1% non-essential amino acids, 100 U/mL penicillin, and 100 µg/mL streptomycin. The Caco-2 cells were subcultured weekly using trypsin-ethylenediaminetetraacetic acid (EDTA), with the medium being refreshed once between passages.

#### 2.6.1. Treatment of Caco-2 Cell Lines with Extract

Experiments were carried out under conventional incubation conditions. The culture medium was substituted with lyophilized pectin extracts, which were dissolved in Hank’s Balanced Salt Solution (HBSS) at a concentration of 100 mg/mL. The solution was then heated to 100 °C for 30 min and subsequently diluted with the culture medium as needed. Cells were plated in 96-well microplates at a density of 4 × 10^4^ cells per well, with 50 μL of medium per well. The extracts were added in equal volumes (50 μL per well) to achieve final concentrations ranging from 1.25 to 10 mg/mL. Caco-2 cells were incubated for up to 7 days.

The assays were additionally conducted on confluent Caco-2 cells, which were seeded in 96-well microplates at a higher density of 14 × 10^3^ cells per well, with 100 μL of medium per well. These cells were allowed to proliferate for two days before being exposed to the samples. Besides the cell viability assessment, the cells were also examined using a phase-contrast microscope (Zeiss Axiovert 25 phase contrast microscope, Zeiss (Oberkochen, Germany)).

#### 2.6.2. Caco-2 Cell Line Proliferation Assays

The proliferation of Caco-2 cells was assessed by measuring cell viability at different time points (1, 3, and 7 days). Confluent Caco-2 cells were similarly evaluated at 3, 4, and 7 days. For the neutral red assay in both cases, cells cultured in 96-well plates were incubated in a fresh culture medium containing neutral red dye (50 μg/mL) for 30 min. Subsequently, cells were washed with HBSS, and the dye was extracted using an acetic acid solution (75 μL, 1% *v*/*v* in 50% *v*/*v* ethanol). The absorbance was then measured at 550 nm using a plate reader (Thermo Fisher Scientific, Waltham, MA, USA), following the method established by Borenfreund and Puerner (1985) [26].

### 2.7. Isolation and Culture of Murine Macrophage Cells

Six female Swiss mice from Janvier Labs^®^ (Le Genest St Isle, France) weighing approximately 20 g were maintained in the animal laboratory facility of the Center for Technology and Innovation Research, University of Seville (CITIUS) (University of Seville) under standard conditions of temperature (24–25 °C), humidity (40–60%), and a 12 h light/dark cycle with free access to food and drink. All animal procedures conformed to the European Union Guidelines and were approved by the Animal Ethics Committee of the University of Seville (ethics approval number 23/07/2018/119). Murine peritoneal macrophages were isolated as previously described by Castejon, 2022 [27].

These macrophages were collected 72 h after intraperitoneal injection with sterile thioglycolate (3.8% *w*/*v*). The collected cells were cultured in the absence and presence of extract at different concentrations (10, 5, and 1 mg/mL) for 30 min and then stimulated with LPS at 5% CO_2_ at 37 °C. The supernatants and cell samples were collected and stored until cytokine measurement.

#### 2.7.1. Murine Peritoneal Macrophages Cell Viability

Sulforhodamine B (SRB) colorimetric assay indicated the viability of macrophages treated with extract. An amount of 1 × 10^5^ cells/mL was cultured in the presence/absence of extract (10, 5, and 1-0.03 mg/mL) for 18 h. The absorbance was read at 520 nm with a microplate reader (BioRad^®^, Hercules, CA, USA). We expressed the absorbance as the percentage of viability when compared to untreated control cells (100% cell survival).

#### 2.7.2. Intracellular Reactive Oxygen Species Detection (DCFDA)

The DCDFA assay kit was performed according to the manufacturer’s instructions (Abcam^®^, Cambridge, UK). DCFDA penetrates into the cells, where it is hydrolyzed by esterases and, with ROS, is oxidized to the highly fluorescent 2′7′-dichlorofluorescein (DFC). Cells (2.5 × 10^5^ cells/mL) were seeded on a black plate, and then we added DCDFA to each well, either previously untreated or treated with extract, and they were stimulated with LPS for 18 h. DCFDA (25 µM) was added to each well at 37 °C for 45 min. Excitation and emission wavelengths (485 and 535 nm, respectively) were read using a fluorescence microplate reader (Biotek^®^, Bad Friedrichshall, Germany).

#### 2.7.3. Evaluation of Anti-Inflammatory Activities

According to our previous works [28], nitrite levels were evaluated by Griess reagent (Sigma-Aldrich^®^, St Louis, MO, USA) at 540 nm using an enzyme-linked immunosorbent assay (ELISA) microplate reader (Biotek^®^, Bad Friedrichshall, Germany). Cells were exposed to varying concentrations of the extract, and after 30 min, they were stimulated with LPS for 18 h. The supernatants were collected and combined with Griess reagent, followed by a 15-min incubation at room temperature. The nitrite levels, serving as an indicator of NO production, were quantified by extrapolating from a standard curve prepared with sodium nitrite. Results were expressed as the nitrite production percentage compared with DMSO-LPS cells (stimulated, untreated cells).

#### 2.7.4. Enzime-Linked Immunosorbent Assay (ELISA)

Cytokine production in the supernatants of peritoneal macrophage cell cultures was assessed using ELISA kits, following the manufacturer’s protocols, to measure TNF-α, IL-β (Peprotech^®^, London, UK), and IL-6 (Diaclone^®^, Besançon Cedex, France). The results were read at 450 nm with an ELISA microplate reader (Biotek^®^, Bad Friedrichshall, Germany). All assays were performed in duplicate.

### 2.8. Statistical Analysis

Results were expressed as mean values ± standard deviation. STATGRAPHICS^®^ Plus software version 19 (Statgraphics Technologies, Inc., The Plains, VA, USA) was used for statistical analysis. Comparisons among samples were made using one-way analysis of variance (ANOVA) and the Least Significant Difference (LSD) method, with a *p*-value < 0.05 considered significant.

Data from murine peritoneal macrophage cell experiments are reported as arithmetic means ± standard error of the mean (SEM) from at least six independent experiments carried out in triplicate. Results were evaluated using GraphPad Prism version 5.01 software (GraphPad Software, San Diego, CA, USA), with statistical significance analyzed by one-way ANOVA, followed by Tukey’s multiple comparisons test. A *p*-value < 0.05 was considered statistically significant.

## 3. Results and Discussion

### 3.1. Preparation of the Pectin–Polyphenol Complex Extracted from Alperujo, Chemical Composition, and Antioxidant Activity

The interaction between pectin and polyphenols is enhanced during olive oil processing [15], significantly impacting their chemical composition. As shown in Table 1, the extract exhibited a relatively low content of uronic acids (15%) and polyphenols (8%), with arabinose and galactose as the predominant neutral sugars in the pectin side chain. While previous studies on similar extracts have achieved a chemical characterization rate exceeding 90% [15], in this instance, only 67% was obtained. As previously mentioned, the composition of these polyphenol-bound pectin extracts is influenced by various factors, including olive variety, growing conditions, fruit maturity, and processing methods, making precise characterization more challenging [12].

**Figure 1:**
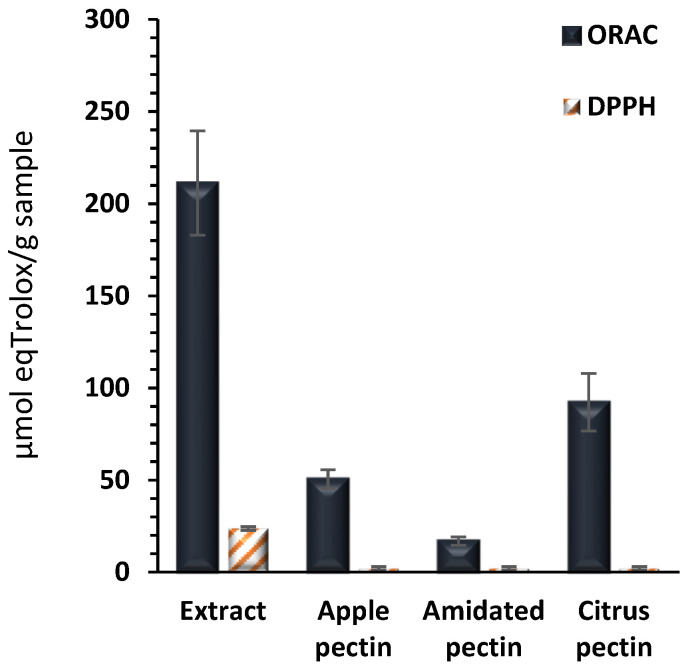
Antioxidant activities, ORAC and DPPH, of extract compared with three commercial pectins. Antioxidant activities are expressed as µmol Trolox equivalent/g extract. Data represent the average of six replicates ± standard deviation.

However, the pectin–polyphenol complex exhibited relatively high antioxidant activity, consistent with previous studies [12,16]. The oxygen radical absorbance capacity (ORAC) and 2,2-Diphenyl-1-picrylhydrazyl (DPPH) radical scavenging activity of the extract are shown in Figure 1. The results indicated an activity of 211 and 24 μmol eq Trolox/g of extract by the ORAC and DPPH assays, respectively. The extract demonstrated 2 to 12 times higher activity than three commercial pectins (apple, amidated, and citrus pectin) by the ORAC method, while none of the commercial pectins showed antiradical DPPH activity at the tested concentrations (up to 20 mg/mL) compared to the complex activity of the extract tested at 5 mg/mL. This underscores the importance of phenolic compounds, which despite being bound to pectin, retain antioxidant activity in vitro. This antioxidant activity may be directly related to antiproliferative and anti-inflammatory activities.

### 3.2. Antiproliferative Activity Studies in Caco-2 Cell Lines

#### Cell Viability Assays in Caco-2 Cell Lines

**Figure 2:**
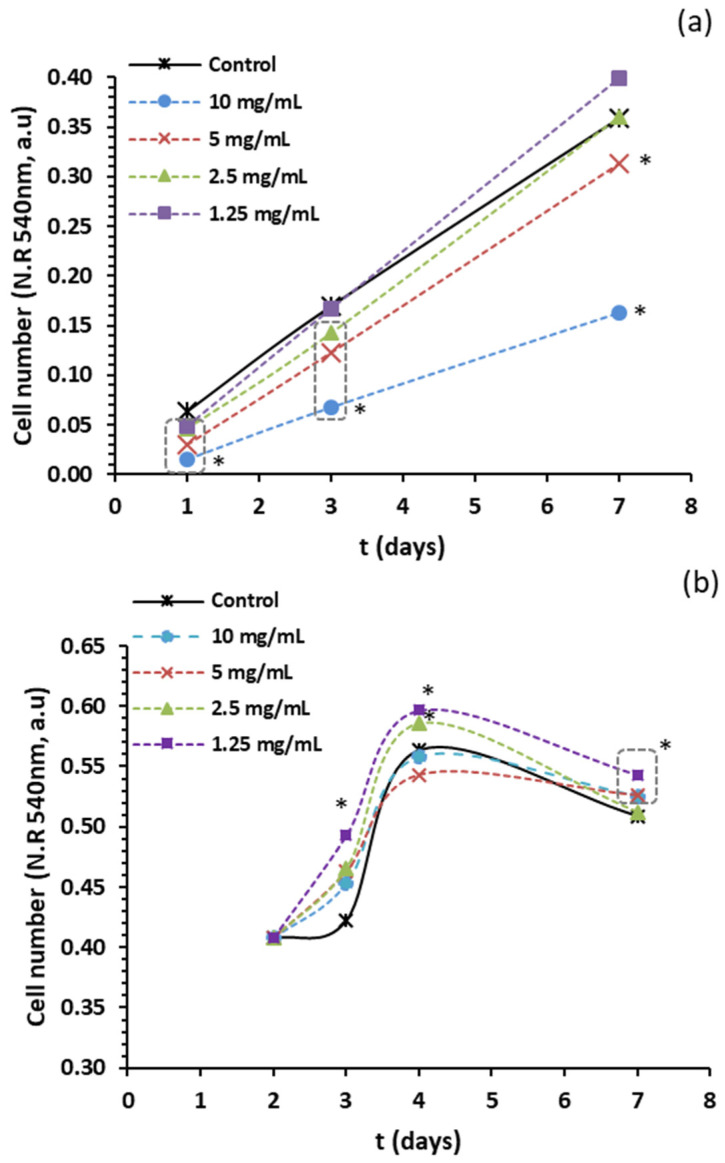
(**a**) Effect of extract on proliferation of Caco-2 cells. Cells (4 × 10^3^ cells per well) were seeded in the presence of increasing concentrations of extract and allowed to proliferate for one to seven days. (**b**) Effect of extract on proliferation of confluent Caco-2 cells. Cells (14 × 10^3^ cells per well) were seeded and incubated for two days. An increasing concentration of the extract was then added, and cells were incubated for up to seven days. Cell number was then estimated by determination of neutral red uptake. Data represent the average of six replicates, and error bars are not shown for clarity. Asterisks indicate a statistically significant difference between treatment and vehicle control for each incubation period (oneway ANOVA-LSD test, *p* < 0.05).

Caco-2 cells, originally isolated from human colon carcinoma, were used as in vitro models to assess the effect of the obtained extract on cell proliferation. The cells were treated with 1.25, 2.5, 5, and 10 mg/mL of the extract for up to 7 days. The lower concentrations (1.25 and 2.5 mg/mL) did not inhibit cell proliferation. However, at 5 mg/mL, we observed an inhibition ranging from 20% to 60%, and at 10 mg/mL, the inhibition ranged from 60% to 80% over all days (Figure 2a). Previous data from our research group indicated that a concentration of 10 mg/mL of the extract resulted in 100% inhibition from the first day [12]. This observed decrease in antiproliferative activity in Caco-2 cell lines could correlate with a reduction in the percentage composition of uronic acid if compared to the extracts obtained in the previous study [12].

Caco-2 cells are used as a model for studying exposure to dietary and drug components [29,30] and as a model of absorption when allowed to differentiate in vitro into an enterocyte-like phenotype [31]. When Caco-2 cells are differentiated in confluent cultures, they become polarized cells that closely resemble healthy enterocytes in both morphology and physiology. Thus, the same cell line can be used to differentiate into transformed cancer cells and healthy epithelial cells. Figure 2b shows the results of exposure of the extracts to confluent Caco-2 cells up to 7 at increasing concentrations of the extracts. This experiment demonstrated that differentiated Caco-2 cells were not adversely affected by the extracts. Notably, a 7-day treatment with extract concentrations ranging from 1.25 to 10 mg/mL resulted in increased cell proliferation compared to untreated cells. Previous research indicated that incubating Caco-2 cells with a complex of pectin and phenols derived from olive oil by-products, as well as with modified citrus pectin (Pectasol), at low concentrations for seven days reversed the inhibition seen with shorter incubation times and actually promoted proliferation [12]. This phenomenon may be related to the fact that Caco-2 cells differentiate towards an enterocytic phenotype when cultured as confluent monolayers, i.e., epithelial cells, and therefore should not be adversely affected by polysaccharide extracts. Therefore, the results of this study suggest that the inhibition of proliferation is dependent on the differentiation status of Caco-2 cells, indicating that this effect is not due to nonspecific toxicity.

### 3.3. Studies of Anti-Inflammatory Activity in Murine Macrophages

#### 3.3.1. Effects of Extract on Cell Viability in Murine Macrophages

**Figure 3:**
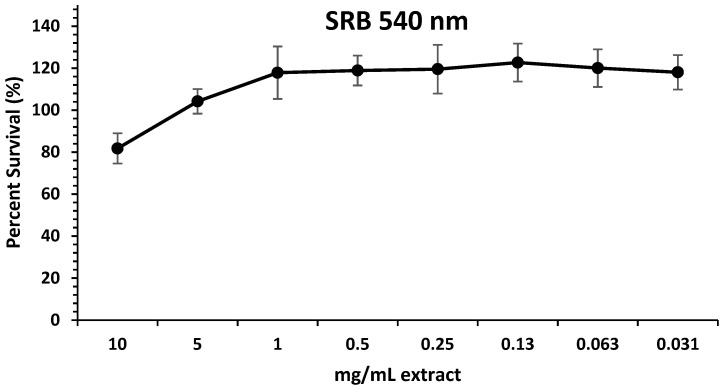
Effect of extract cell viability. Macrophages were pretreated with extract (0.03125–10 mg/mL) for 18 h. Cell survivals were expressed as the percentage of viability with respect to 100% from control, untreated cells. Results are presented as mean ± SEM of at least six independent experiments.

The macrophages are the main components of the innate immune system, and they can modulate inflammatory and immune responses. For the first time, we analyzed the impact of the extract on murine peritoneal macrophages by SRB survival assay. After 18 h of treatment with compounds, the viability of cells treated with this was not significantly compromised at the concentrations studied (Figure 3). So, we decided to assay the compounds at concentrations 10, 5, and 1 mg/mL in forthcoming experiments. Furthermore, this would confirm that the effect of the extracts is not due to nonspecific toxicity, as previously observed in Caco-2 and differentiated Caco-2 cells, since it would not compromise the viability of murine peritoneal macrophages.

#### 3.3.2. Extract Downregulated Intracellular ROS and Nitrite Production in LPS-Stimulated Murine Peritoneal Macrophages

**Figure 4:**
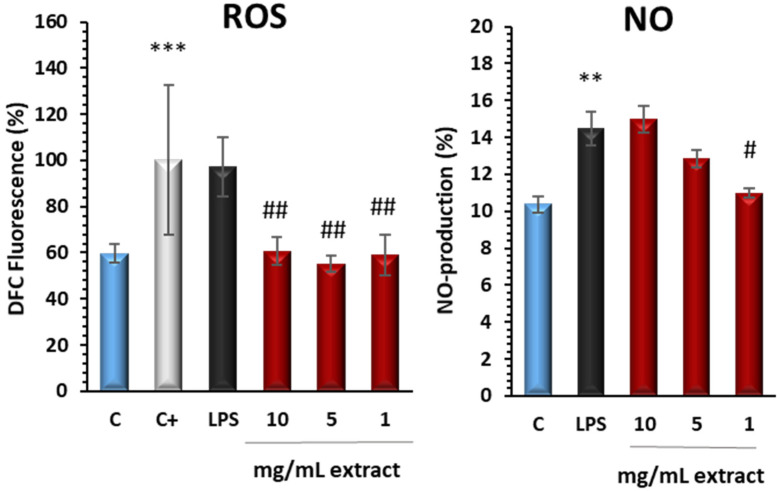
Effects of extract on intracellular ROS and NO production were measured using DCFDA and Griess assays in supernatants, respectively. Macrophages were pretreated with extract (10, 5, and 1 mg/mL) for 30 min and then LPS-stimulated for 18 h. Subsequently, ROS and NO levels. The positive control (C+) represents the maximum ROS production achieved by treating the cells with hydrogen peroxide (H_2_O_2_). Results are presented as the mean ± SEM of at least six independent experiments. ** *p* < 0.01; *** *p* < 0.001 vs. unstimulated control cells; ^#^ *p* < 0.05; ^##^ *p* < 0.01; vs. LPS stimulated cell.

As a result of the imbalance in cellular redox homeostasis, intracellular oxidative stress primarily manifests as ROS-mediated damage. This oxidative stress induces the upregulation of the iNOS gene, leading to increased production of nitric oxide (NO), among other effects [32]. In experiments with murine peritoneal macrophages stimulated with LPS, a significant increase in pro-inflammatory mediators was observed. Our findings demonstrated that treatment with the extract reduced intracellular ROS levels at concentrations of 10, 5, and 1 mg/mL and decreased NO production, specifically at 1 mg/mL (Figure 4). Upon closer examination of LPS-induced murine peritoneal macrophages, we noted a reduction in NO levels in cell supernatants following extract treatment. This reduction in NO generation is likely linked to the antioxidant properties of the extract. NO, a free radical, is implicated in oxidative stress and acts as a pro-inflammatory mediator in both acute and chronic inflammation. Furthermore, our DPPH and ORAC assays confirmed significant antioxidant activity in the extract, higher than commercial pectins, likely due to its phenolic compound content, which also explains the observed decrease in ROS. Studies such as those by Boukhers et al. (2023) [33] on orange flesh sweet potato (OFSP) flour highlight the substantial antioxidant activity of these compounds. OFSP flour, rich in fiber, minerals, beta-carotene, and polyphenols, has demonstrated the ability to scavenge free radicals and reduce the production of several pro-inflammatory cytokines, including NO and IL-6, in murine macrophages.

#### 3.3.3. Effects of Extract on Cytokines Secretion

**Figure 5:**
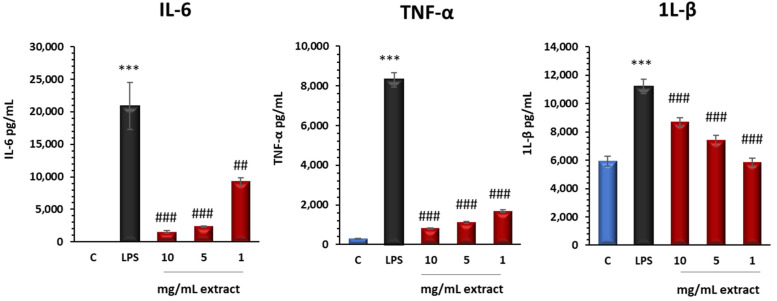
Pro-inflammatory cytokine levels were downregulated in extract-treated cells. IL-1β, IL-6, and TNF-α levels were measured by ELISA on cell supernatants. Macrophages were pretreated with extract (10, 5, and 1 mg/mL) for 30 min and then LPS-stimulated for 18 h. Results are presented as the mean ± SEM of at least six independent experiments. *** *p* < 0.001 vs. unstimulated control cells; ^##^ *p* < 0.01; ^###^ *p* < 0.001 vs. LPS stimulated cell.

In order to analyze the anti-inflammatory activity ex vivo, tests were conducted using LPS, a lipopolysaccharide from *Escherichia coli*, which induces inflammation in murine peritoneal macrophages by prompting them to secrete pro-inflammatory cytokines (TNF-α, IL-6, and IL-1β). The release of these cytokines is a primary indicator of the inflammatory state. If the extract inhibits the gene expression of any pro-inflammatory cytokine, it could be considered a potential therapeutic agent for combating inflammation. The stimulation of murine peritoneal macrophages with LPS triggers a cascade of immune responses characterized by the dysregulation of pro-inflammatory and anti-inflammatory cytokines. Previous studies by researchers such as Castejón et al. (2019) [27] and Aparicio-Soto et al. (2015) [34] have documented this phenomenon, demonstrating a significant increase in pro-inflammatory cytokines, including IL-1β, TNF-α, and IL-6, upon LPS exposure. The impact of plant polysaccharides on cytokine production by macrophages suggests their potential as immunomodulators, with either anti-inflammatory or pro-inflammatory applications [35,36]. In agreement with these observations, the present results revealed that IL-1, IL-6, and TNF- levels were increased after 18 h of LPS activation. However, treatment with the extract at concentrations of 10, 5, and 1 mg/mL significantly reduced the levels of these cytokines compared with the macrophages with only LPS treatment, indicating a strong anti-inflammatory potential of the extract (Figure 5).

Different authors have indicated that pectic polysaccharides can reduce the production of pro-inflammatory cytokines [37,38]. Moreover, different monosaccharide compositions and molecular weights have been found to enhance the immunostimulant activity of pectin oligosaccharides compared to whole pectin [39]. In fact, TNF-α release plays an important role in inflammation during the acute phase and promotes inhibition of tumor cell division and macrophage-mediated tumor cytotoxicity mechanisms [40]. The anti-inflammatory efficacy of the extract is underscored by its ability to downregulate TNF-α production, essential for inflammation during the acute phase, and inhibition of tumor cell division and macrophage-mediated tumor cytotoxicity mechanisms. As shown in Figure 5, TNF-α production decreased after treatment of macrophages with the extract at all concentrations tested compared to LPS. On the other hand, IL-6 is a critical cytokine involved in the immune response, inflammation, and hematopoiesis. It plays a key role in the regulation of immune responses and is produced by various cell types, including T cells, macrophages, and endothelial cells. IL-6 can have both pro-inflammatory and anti-inflammatory effects depending on the context of its production and the receptors involved [41]. In Figure 5, it is evident that IL-6 production decreased following macrophage treatment with the extract across all tested concentrations, contrasting with LPS stimulation. Similar outcomes were observed in comparative studies, indicating that IL-6, at the same as TNF-α, is subject to modulation by diverse extracts and natural compounds, thus emphasizing its significance in inflammation and immune response regulation [42,43,44]. As shown in Figure 5, the decreasing level of IL-1β underlines the importance of the extracts in the anti-inflammatory activity since this cytokine is involved in IL-1β, another pro-inflammatory cytokine that exerts pleiotropic effects on a variety of cells and plays key roles in acute and chronic inflammatory and autoimmune disorders. This cytokine is a potent pro-inflammatory agent crucial for host defense responses to infection and injury. Overproduction of IL-1β is implicated in the pathophysiological changes occurring in various disease states, such as rheumatoid arthritis, pain, inflammatory bowel disease, osteoarthritis, multiple sclerosis, and Alzheimer’s disease, among others [45].

Upon closer examination of LPS-induced murine peritoneal macrophages, we observed a simultaneous decrease in nitric oxide (NO) levels as well as pro-inflammatory cytokines. This correlation suggests a multifaceted mechanism of action whereby the extract not only directly influences cytokine production but also indirectly attenuates inflammation-associated oxidative damage through the regulation of NO levels. By maintaining cellular homeostasis through modulation of these key inflammatory mediators, the extract demonstrates its potential as a comprehensive anti-inflammatory agent with implications for therapeutic intervention in inflammatory diseases. Therefore, it is relevant to study the role of our extract in the modulation of these types of cytokines.

These observations suggest that the extract has significant anti-inflammatory potential, comparable to other known modulators of these cytokines, likely linked to its high antioxidant activity, as confirmed by our DPPH and ORAC assays. This opens new perspectives for its therapeutic use in inflammatory and autoimmune diseases.

## 4. Conclusions

This study successfully extracted pectin–polyphenol extract from olive oil by-products using citric acid treatment at 80 °C, highlighting its significant chemical composition and antioxidant activity, demonstrating higher antioxidant activity than commercial pectin. This study highlights the potential of using alperujo, a common olive oil by-product, as a valuable source of bioactive compounds.

The extract exhibited notable antiproliferative effects in Caco-2 cell lines, selectively inhibiting cancer cell proliferation while sparing healthy, differentiated cells. This selective cytotoxicity suggests potential applications in cancer therapy, where targeted inhibition of tumor cells could be beneficial. Additionally, the extract’s ability to reduce intracellular ROS and NO production in LPS-stimulated murine macrophages highlights its potential for modulating oxidative stress and inflammatory responses, with significant downregulation of pro-inflammatory cytokines IL-1β, TNF-α, and IL-6 indicating its potential for managing inflammation-related conditions.

To build on these findings, future research should focus on optimizing the extraction processes, exploring the bioavailability and metabolism of the active compounds, and conducting in vivo studies to further validate the therapeutic potential of this extract. Moreover, investigating practical methods for utilizing or delivering this by-product to potential consumers, such as incorporating it into commercial health supplements or agricultural products, could enhance its application and economic value. Exploring these avenues could further establish the utility of alperujo-derived compounds in both health and industry.

Overall, this research contributes valuable insights into the antioxidant and anti-inflammatory properties of pectin–polyphenol complexes and suggests promising directions for future studies and practical applications of olive oil by-products.

## Figures and Tables

**Table 1:** The chemical composition (g/100 g) and glycosyl residue composition (% molar ratio) of extracts obtained through thermal processing at 80 °C with citric acid are presented. The data are expressed as mean ± standard deviation.

	g/100 g Extract
Uronic acid	16 ± 1.2
Phenols	7.9 ± 0.19
Neutral sugars	34 ± 0.91
Ash	9.1 ± 1.6
	% molar
Rhamnose	0.91
Fucose	0.14
Arabinose	33
Xylose	4.9
Mannose	4.3
Galactose	20
Glucose	5.3
Uronic acid	32

## Data Availability

The data presented in this study are available in the article.
